# Serum concentrations of Krebs von den Lungen-6 as prognostic biomarker in patients with silicosis

**DOI:** 10.3389/fmed.2025.1579209

**Published:** 2025-06-06

**Authors:** Raluca-Andreea Smărăndescu, Marina-Ruxandra Oțelea, Eugenia Panaitescu, Andrä Jitka, Francesco Bonella, Agripina Rașcu

**Affiliations:** ^1^Carol Davila University of Medicine and Pharmacy, Clinical Department 5, Internal Medicine, Bucharest, Romania; ^2^Department of Occupational Medicine, Colentina Clinical Hospital, Bucharest, Romania; ^3^Carol Davila University of Medicine and Pharmacy, Department 3 - Complementary Sciences, Bucharest, Romania; ^4^Center for Interstitial and Rare Lung Disease, Department of Pneumology, Ruhrlandklinik University Hospital, University of Duisburg-Essen, Essen, Germany

**Keywords:** KL-6, silicosis, occupational fibrosis, crystalline silica, biomarker, disease progression

## Abstract

**Introduction:**

KL-6, a pneumocyte derived biomarker, is increased in patients with interstitial lung diseases (ILDs). We aimed to investigate the role of serum KL-6 as a diagnostic and prognostic biomarker in silica-exposed workers.

**Material and method:**

We studied 108 silica-exposed subjects and 25 healthy controls. Chest radiography (CXR), pulmonary function tests, inflammatory markers were collected. Progressive massive fibrosis (PMF) was defined according to the ILO classification. KL-6 was measured in serum by fully automated CLEIA at first presentation at our institution, intended as baseline visit, with a time point from the initial exposure variable for each patient.

**Results:**

PMF was present in 23 subjects. Serum KL-6 levels were significantly higher in subjects with PMF than in controls, exposed workers or simple silicosis (*p*<0.001). In PMF, serum KL-6 positively correlated with C-reactive protein (CRP) and Erythrocyte sedimentation rate (ESR), and negatively with forced vital capacity (FVC) % predicted. At a cut-off of 436 U/mL, serum KL-6 differentiated exposed workers from PMF with a specificity and sensitivity exceeding 90% (*p*<0.0001), while a cut-off of 445 U/mL differentiated simple silicosis from PMF (*p*<0.0001). In the multivariate analysis, serum KL-6 levels were independently associated with risk of fibrosis.

**Conclusion:**

Serum KL-6 appears to be a promising biomarker for the occurrence and progression of PMF in silica-exposed workers.

## Introduction

Pneumoconioses represent a group of chronic lung disorders characterized by pulmonary fibrosis, often as a result of occupational exposure to respirable dust or mineral fibers. Respiratory exposure in the workplace environment to pneumoconiogenic minerals, precipitate their accumulation within lung tissues, eliciting tissue responses characterized by inflammation and variable extension of fibrosis (1).

The fibrogenic potential of mineral powders depends on their physical and chemical properties, lung retention, and the individual’s physiological and immunological traits (2). Accumulation of particles in the lungs can trigger a self-sustaining inflammatory process, leading to disease progression even after exposure has ceased, typically following at least 5 years of occupational exposure (2–5). Data on the dust quantities triggering inflammation in humans remains insufficient, though studies in mice indicate that 0.2 mg of retained dust might be enough (5, 6). Thus, exposure limit values and exposure time are only guidelines and do not guarantee protection for all individuals (7).

Despite all the preventive measures implemented in the last decades (8), pneumoconioses still represent a public health concern, both globally and in Romania (8, 9). Studies over the past decade by the Global Burden of Disease (GBD) Center indicate a downward trend in pneumoconioses prevalence, yet approximately 60,000 new cases were confirmed in 2017, with a global prevalence of around 527,500 cases (9).

In accordance with the ILO (International Labor Office) recommendations (10), the diagnosis of pneumoconioses adheres to fundamental criteria, including occupational exposure history, symptomatology, clinical signs, respiratory functional changes, radiological findings compatible with pneumoconiosis, and exclusion of mimicking conditions (10, 11). However, as in other occupational lung diseases, the histopathological onset often precedes early radiological and clinical manifestations (11). The identification of biomarkers for (1) early fibrosis diagnosis, (2) disease severity assessment, (3) progression and survival prognostication is still an unmet need (12).

In light of this, Krebs von den Lungen-6 (KL-6) seems to fulfill the above criteria. KL-6, also known as Mucin-1 (MUC-1), is a circulating high-molecular-weight mucin-like glycoprotein (13, 14), predominantly expressed in the cytoplasm and membrane of type II pneumocytes (15, 16), and in bronchial epithelial cells, particularly Clara cells (non-ciliated bronchiolar secretory cells) (17, 18). Previous research indicates that the elevated level of KL-6 in bronchoalveolar lavage fluid (BALF) in idiopathic pulmonary fibrosis (IPF) reflects the proliferation of type II pneumocytes, which are also stimulated by the exposure to crystalline silica dust (19). Furthermore, serum KL-6 levels correlate with disease severity in different pulmonary conditions (20) and this correlation may also extend to pulmonary fibrosis resulting from exposure to dust and mineral fibers.

The objective of this study was to investigate whether serum KL-6 could have a role as a diagnostic/prognostic marker in exposed workers to crystalline silica dust to predict worse disease outcome.

## Patients and methods

### Study population and study design

Between 2022 and 2023, 120 subjects with exposure to mineral dust or already diagnosed with pneumoconiosis were admitted in the Occupational Medicine Clinic of Colentina Clinical Hospital, Bucharest, Romania. A detailed anamnesis was performed for all patients and all had a documented occupational exposure of at least 5 years to pneumoconiogenic dusts.

As the current study was dedicated to silicosis, all patients diagnosed with other forms of occupational pulmonary fibrosis (10 patients with asbestosis and 2 with kaolinosis) were excluded and 108 were included in the further analysis. As the prevalence of PMF in the literature ranges from 7.5 to 10% (21, 22), we estimated a sample size between 107 and 139 patients necessary for the purpose of this study.

None of the patients was less than 18 years old and all participants signed an informed consent for inclusion and data processing. Patients with contraindications for pulmonary function tests according to the current international guidelines (23) were also excluded. The study obtained approval from the Ethics Committee of Carol Davila University of Medicine and Pharmacy under protocol No. 8244/28.03.2022.

Clinical and paraclinical investigations were conducted subsequent to consent acquisition.

This retrospective study included 108 subjects who were former or actual cast iron foundry workers, involved in activities such as: abrasive cutting, sand casting, mold dislodgement, cast iron polishing, and furnace maintenance. None of them was miner, as Bucharest in not located in a mining area and there are very few miners with silicosis who are surveilled in this Occupational Medicine Clinic. Given the homogeneity of their professional background, which ensured similar exposure intensity, the primary factors differentiating their exposure were the duration of exposure and retention time. The 108 subjects were monitored for an average duration of 13 years (13.29 ± 5.92), with no statistically significant differences between subgroups. The diagnosis of silicosis was determined based on occupational history, a minimum exposure of 5 years, clinical symptoms, and imaging findings.

As control group, 25 subjects with no occupational exposure to chemical, dust, fibers or biological agents and no medical history of lung disease were selected. In accordance with national regulations, all case group members underwent standard chest radiographs (CXRs) assessed by the Pneumoconiosis Board, aligned with the 2022 revised edition of the ILO Guidelines (10).

### Clinical definitions

PMF was defined as complicated silicosis with large opacities (greater than 1 cm) on CXR according to the ILO categories A, B, C [Appendix A]. The following definitions were used: exposure time as effective duration of exposure, retention time as period of time the pneumoconiogenic dusts and fibers were retained in the lungs, and latency time as period from the initial day of exposure to the first diagnosis of the disease. The durations of exposure, retention, and latency were reported in years (3).

### Occupational dust exposure: assessment protocol

A standardized questionnaire was applied to all subjects in order to collect data regarding: occupational exposure, occupational history, environmental history, demographic data, and medical background. The questionnaire was administered for both the study and control groups. Its primary objective was to select patients with occupational exposure to crystalline silica dust to be included in the study group and to exclude silica and other occupational respiratory hazards in the control group. The questionnaire was meticulously structured, comprising multiple sections tailored to comprehensively capture occupational and medical histories [Appendix B].

### Radiographic evaluation

CXR served as the modality to stratify individuals within the case group (those occupationally exposed to crystalline silica dust) in exposed without silicosis, patients with simple silicosis and patients with complicated silicosis. The classification followed the International Labour Office Guidelines on the International Classification of Radiographs of Pneumoconiosis: simple pneumoconiosis was characterized by small opacities ≤ 1 cm and complicated pneumoconiosis (progressive massive fibrosis) was defined by the presence of larger opacities exceeding 1 cm. Every radiographic image underwent scrutiny by the Pneumoconioses Board, comprising occupational medicine physicians and radiologists with long-term experience in diagnosing occupational pulmonary fibrosis.

### Blood tests: laboratory procedures

Venous blood samples were obtained from each patient and stored at −20°C until measurement.

KL-6 serum levels were quantified using Fujirebio Lumiplse G600II through automated chemiluminescent enzyme immunoassay (CLEIA) as previously described (24) employing a two-step immunoassay methodology. The intra-assay coefficient of variation was 2.7% and the inter-assay coefficient of variation was 3.1%, according to the package insert Lumipulse G KL-6 Immunoreaction Cartridges (key-code: FRI87290), Fujirebio Inc. KL-6 serum levels were expressed in U/mL.

### Pulmonary function tests

All participants included in the study (*N* = 133) underwent spirometry to evaluate their lung function with a MasterScreen Pneumo PC spirometer, CareFusion. Spirometric assessments adhered to the guidelines outlined by the American Thoracic Society (ATS) and were performed by experienced practitioners within the Pulmonary Function Lab of the Occupational Medicine Clinic, Colentina Clinical Hospital.

### Statistical analysis: methodology

To assess the normality of variables, the Kolmogorov–Smirnov and Shapiro–Wilk tests were employed. Variables demonstrating a normal distribution are presented as mean ± standard error of the mean (SEM), while those with non-normal distribution are depicted as median with interquartile range (IQR). Parametric tests were applied to variables with normal distribution, whereas nonparametric tests were utilized for data exhibiting abnormal distribution. Specifically, ANOVA was employed for quantitative variables with normal distribution, while the Kruskall-Wallis and Mann–Whitney U tests were utilized for data with abnormal distribution. For qualitative variables, Pearson Chi-Square, Likelihood Ratio, and Fisher’s exact test were employed for data comparison. To compare variables between subgroups, we used *post hoc* tests and made the adjustment for multiple testing using Bonferroni correction.

Pearson’s correlation coefficient was used to analyze the associations between serum KL-6 levels and several covariates, such as pulmonary function parameters and inflammatory markers.

To evaluate the role of serum KL-6 as a progression biomarker in silicosis, ROC curve analysis was conducted. The analysis reported the area under the curve (AUC), 95% confidence interval, and *p*-value [Significance level P (Area = 0.5)]. Cut-off values were determined with a minimum requirement of a sensitivity and specificity of at least 90%. Univariate and multivariate Cox proportional hazard regression models were employed to assess prognostic factors for silicosis occurrence and progression to PMF. The Kaplan–Meier method with the log-rank test, was utilized to determine the association between KL-6 serum levels and disease outcome. A *p*-value < 0.05 was considered statistically significant. All statistical analysis was conducted using IBM SPSS Statistics software, version 26.0 (SPSS Inc., Chicago, IL, USA).

## Results

### Characteristics of study subjects

The demographic characteristics, occupational history, and medical backgrounds of the enrolled subjects are detailed in Table 1. Based on CXR outcomes and occupational history, the case group was stratified into distinct subgroups: 21 exposed workers, without silicosis (EWs), 64 simple silicosis subjects, with opacities <1 cm (SSs), and 23 complicated silicosis subjects, progressive massive fibrosis with opacities >1 cm (CSs).

**Table 1:** Clinical, paraclinical and demographic characteristics of the subjects.

	EWs	SSs	CSs	HCs	*p*-value
Demographics, occupational history, and medical background					
N	21	64	23	25	
Age (years)	53.0 [50.5, 57.5]	56.0 [54.0, 65.5]	59.0 [55.0, 65.0]	57.0 [53.5, 61.5]	0.502
Female:Male	3:18	27:37	5:18	9:16	0.066
Exposure time (years)	15.0 [12.0, 22.0]	19.0 [14.0, 24.75]	19.0 [14.0, 29.0]	N/A	0.207
Retention time (years)	31.0 [23.5, 38.5]	36.0 [33.25, 40.75]	37.0 [33.0, 46.0]	N/A	**0.008**
Smoker	10/21 (47.6%)	33/64 (51.6%)	6/23 (26.1%)	3/25 (12%)	**0.003**
Alcohol	7/21 (33.3%)	15/64 (23.4%)	4/23 (17.4%)	8/25 (32%)	0.312
Other occupational lung diseases, of which:	13/21 (61.9%)	50/64 (78.1%)	17/23 (73.9%)	0/25 (0%)	**<0.001**
Occupational lung cancer	0/21 (0%)	1/64 (1.6%)	2/23 (8.7%)	0/25 (0%)	0.137
Occupational asthma	1/21 (4.8%)	3/64 (4.7%)	1/23 (4.3%)	0/25 (0%)	0.751
Occupational chronic bronchitis	12/21 (57.1%)	46/64 (71.9%)	16/23 (69.6%)	0/25 (0%)	**<0.001**
Hypertension	10/21 (47.6%)	42/64 (65.6%)	14/23 (60.9%)	9/25 (36%)	0.063
Diabetes	3/21 (14.3%)	12/64 (18.8%)	4/23 (17.4%)	1/25 (4%)	0.364
Coronary heart disease	4/21 (19%)	23/64 (35.9%)	8/23 (34.8%)	4/25 (16%)	0.180
BMI	27.4 [23.5, 32.3]	28.53 [25.54, 32.83]	26.78 [23.94, 27.41]	29.4 [26.2, 34.7]	**0.023**
Pulmonary function test results					
VC (L)	4.42 [3.86, 4.96]	3.08 [2.66, 3.74]	3.32 [2.36, 4.28]	3.82 [3.42, 4.49]	**<0.001**
VC, % pred	91.8 [85.4, 106.5]	91.10 [82.70, 101.0]	82.90 [70.60, 101.0]	93.9 [84.7, 99.54]	0.252
FVC (L)	4.14 ± 1.104	3.08 ± 0.758	3.10 ± 1.084	3.72 ± 0.707	**<0.001**
FVC, %pred	96.47 ± 24.215	89.09 ± 14.729	79.33 ± 19.925	90.90 ± 13.663	**0.014**
FEV1 (L)	3.58 [2.87, 3.98]	2.40 [1.98, 2.88]	2.20 [1.52, 3.04]	3.23 [2.77, 3.77]	**<0.001**
FEV1, %pred	95.05 [84.65, 110.3]	87.20 [75.15, 97.55]	74.90 [52.30, 98.20]	98.65 [88.55, 104.3]	**<0.001**
FEV1/FVC (%)	81.83 [74.90, 84.98]	81.54 [74.48, 85.62]	74.36 [61.76, 83.96]	87.01 [82.10, 91.01]	**<0.001**
FEF50 (L/s)	3.94 [2.16, 4.87]	2.56 [1.99, 3.34]	1.92 [0.80, 2.88]	3.98 [3.57, 4.83]	**<0.001**
FEF50, %pred	82.3 [52.7, 106.8]	64.30 [49.60, 80.30]	46.50 [18.00, 72.50]	91.6 [85.0, 106.3]	**<0.001**
Inflammatory markers and KL-6 serum concentrations					
Fibrinogen (mg/dL)	298.0 [278.5, 387.5]	374.5 [310.8, 404.8]	366.0 [310.0, 394.0]	305.0 [258.0, 350.0]	**0.001**
CRP (mg/L)	1.98 [1.54, 5.58]	2.83 [1.39, 5.58]	2.41 [1.09, 5.48]	3.28 [2.08, 4.95]	0.871
ESR (mm/1 h)	13.00 [7.50, 27.00]	20.50 [14.00, 29.75]	20.00 [14.00, 41.00]	13.00 [9.50, 14.50]	**0.001**
LDH (U/L)	210.0 [200.0, 231.0]	207.5 [179.3, 242.0]	211.0 [185.0, 241.5]	187.0 [145.0, 227.5]	0.112
KL-6 (U/mL)	299.0 [271.5, 338.0]	317.0 [244.5, 506.5]	532.0 [483.0, 750.0]	309.0 [235.0, 392.5]	**<0.001**

Variables with a normal distribution are presented as mean ± standard error of the mean (SEM), while those with non-normal distribution are depicted as median with interquartile range [IQR]. EWs, exposed workers; SSs, simple silicosis subjects; CSs, complicated silicosis (progressive massive fibrosis) subjects; HCs, healthy controls; BMI, body mass index; VC, vital capacity; FVC, forced vital capacity; FEV1, forced expiratory volume in the first second; FEV1/FVC, the Tiffeneau-Pinelli index; FEF50%, maximal expiratory flow at 50% of the forced vital capacity; CRP, C-reactive protein; ESR, Erythrocyte sedimentation rate; LDH, Lactate dehydrogenase; KL-6, Krebs von den Lungen-6.

Differences between subgroups: Retention time: EWs-SSs (*p* = 0.024), EWs-CSs (*p* = 0.003); Smoker: SSs-HCs (*p* = 0.003); Chronic bronchitis: EWs-HCs (*p* = 0.000), SSs-HCs (*p* = 0.000), CSs-HCs (*p* = 0.000); BMI: no significant differences between subgroups; VC(L): SSs-HCs (*p* = 0.018), EWs-SSs (*p* = 0.000), EWs-CSs (0.031); FVC(L): EWs-SSs (*p* = 0.000), EWs-CSs (*p* = 0.000), SSs-HCs (*p* = 0.004), CSs-HCs (*p* = 0.019); FVC, %pred: EWs-CSs (*p* = 0.002), SSs-CSs (*p* = 0.025), CSs-HCs (*p* = 0.029); FEV1 (L): EWs-SSs (*p* = 0.000), EWs-CSs (*p* = 0.002), SSs-HCs (*p* = 0.001), CSs-HCs (*p* = 0.004); FEV1, %pred: CSs-HCs (*p* = 0.012); FEV1/FVC (%): EWs-HCs (*p* = 0.041), SSs-HCs (*p* = 0.002), CSs-HCs (*p* = 0.000); FEF50 (L/s): EWs-CSs (*p* = 0.005), SSs-HCs (*p* = 0.000), CSs-HCs (*p* = 0.000); FEF50, %pred: EWs-CSs (*p* = 0.025), SSs-HCs (*p* = 0.000), CSs-HCs (*p* = 0.000); Fibrinogen (mg/dL): SSs-HCs (*p* = 0.001), CSs-HCs (*p* = 0.036); ESR (mm/1 h): SSs-HCs (*p* = 0.002), CSs-HCs (*p* = 0.013); KL-6 (U/mL): EWs-CSs (*p* = 0.000), SSs-CSs (*p* = 0.000), CSs-HCs (*p* = 0.000). Statistically significant p-values (*p* < 0.05) are shown in bold.

### Pulmonary function test results

Table 1 illustrates the pulmonary function parameters. Significantly differences were observed among the groups in terms of *FVC % pred*. *Between*: EWs and CSs (*p*-value = 0.001), SSs and CSs (*p*-value = 0.023), CSs and HCs (*p*-value = 0.027); *FEV1% pred. Between:* CSs and HCs (*p*-value = 0.005), CSs and EWs (*p*-value = 0.031); *the FEV1/FVC ratio between*: EWs and HCs (*p*-value = 0.026), SSs and HCs (*p*-value = 0.001), CSs and HCs (*p*-value < 0.001); *FEF50% pred. Between*: EWs and CSs (*p*-value = 0.011), SSs and HCs (*p*-value <0.001), CSs and HCs (*p*-value < 0.001).

### Inflammatory markers and KL-6 serum concentrations

**Figure 1:**
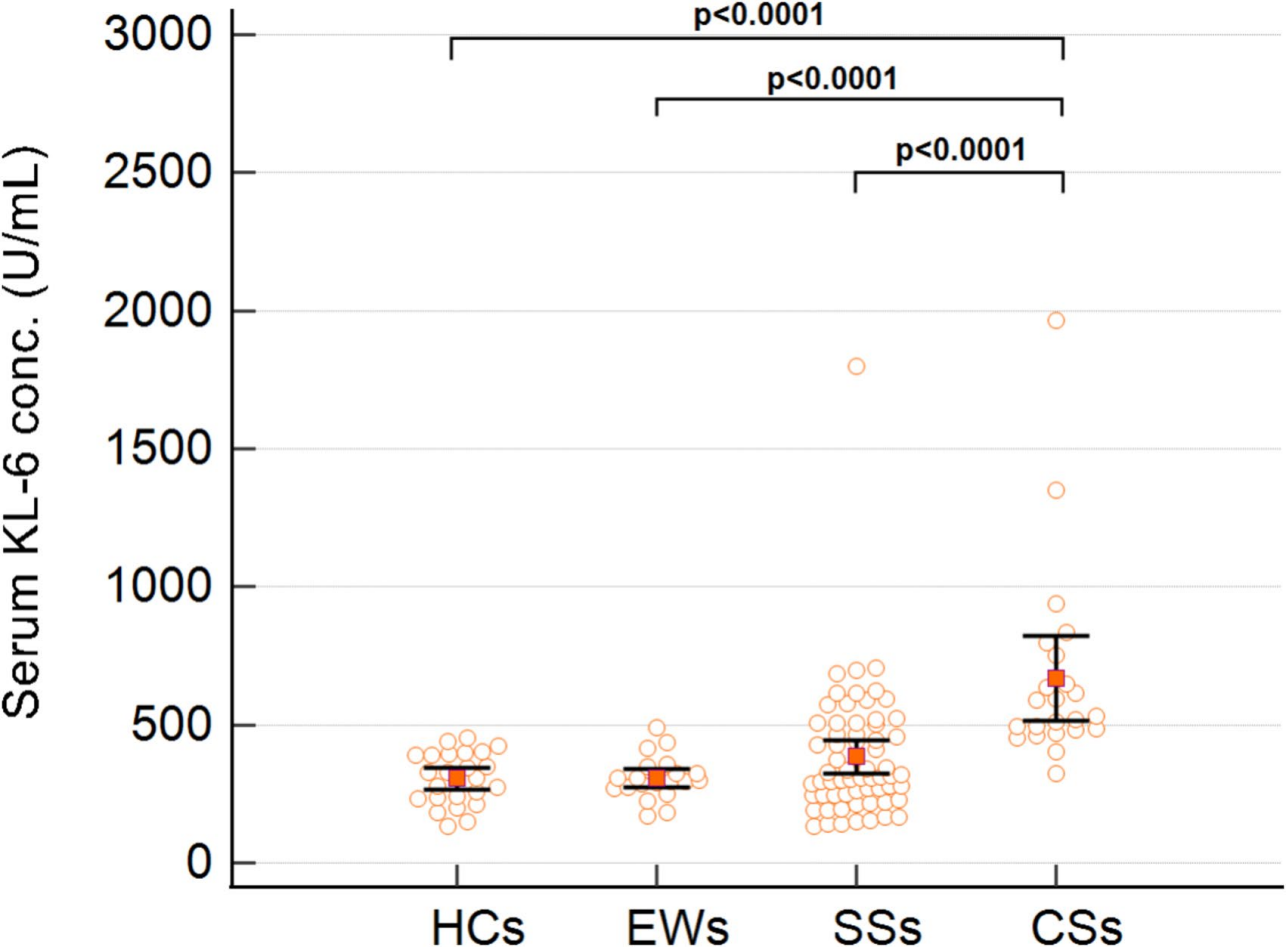
Serum KL-6 levels in the healthy controls (HCs), exposed workers (EWs), simple silicosis (SSs) and complicated silicosis (CSs) groups. White dot: patient; orange square: median; black lines: interquartile range.

Non-specific inflammatory markers assessed across subject groups are shown in Table 1. Significant differences (*p*-value < 0.05) were noted in Fibrinogen and ESR levels across the SSs, CSs, and HCs groups, with the following findings: (1) Fibrinogen: HCs and SSs, HCs and CSs; (2) ESR: HCs and SSs, HCs and CSs. Conversely, no significant variations were discerned in serum CRP and LDH concentrations among subjects across the four groups. Significant differences (*p*-value < 0.001) were noted in KL-6 serum concentrations (Figure 1) across the silicosis stages: EWs and CSs, SSs and CSs, and CSs and HCs.

### Correlations between KL-6 serum concentrations, inflammatory markers and pulmonary function tests among SSs and CSs groups

For the SSs group, there were no correlations between serum KL-6 levels, inflammatory markers, or pulmonary function test parameters, as indicated in Table 2. In the CSs group, KL-6 serum levels were positively correlated with CRP and ESR, and negatively correlated with VC, % pred., and FVC, % pred., as shown in Table 2 and Appendix C.

**Table 2:** KL-6 correlations with inflammatory markers and pulmonary function test results.

		Fibrinogen	CRP	ESR	LDH	VC, % predicted	FVC, % predicted	FEV1, % predicted	FEV1/FVC	FEF50, % predicted
KL-6 in SSs group	Pearson Correlation	0.049	0.133	0.054	−0.111	−0.143	−0.132	−0.058	0.038	−0.041
	Sig. (2-tailed)	0.700	0.294	0.672	0.398	0.270	0.312	0.658	0.774	0.755
	N	64	64	64	60	61	61	61	61	61
KL-6 in CSs group	Pearson Correlation	0.212	0.447^*^	0.439^*^	0.308	−0.544^**^	−0.467^*^	−0.389	−0.257	−0.116
	Sig. (2-tailed)	0.332	0.033	0.036	0.175	0.007	0.025	0.067	0.237	0.598
	N	23	23	23	21	23	23	23	23	23

** Correlation is significant at the 0.01 level (2-tailed); * Correlation is significant at the 0.05 level (2-tailed). KL-6, Krebs von den Lungen-6; SSs, simple silicosis subjects; CSs, complicated silicosis (progressive massive fibrosis) group; CRP, C-reactive protein; ESR, Erythrocyte sedimentation rate; LDH, Lactate dehydrogenase; VC, vital capacity; FVC, forced vital capacity; FEV1, forced expiratory volume in the first second; FEV1/FVC, the Tiffeneau-Pinelli index; FEF50%, maximal expiratory flow at 50% of the forced vital capacity.

### Serum KL-6 cut-off values and ROC curve analysis

**Figure 2:**
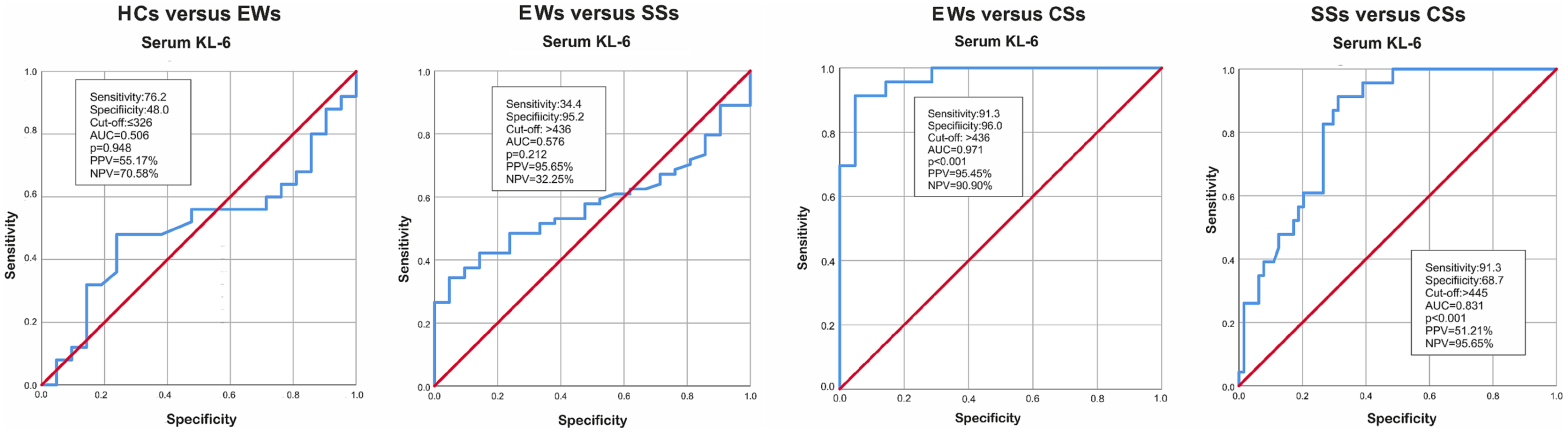
AUC for HCs versus EWs, EWs versus SSs, EWs versus CSs and SSs versus CSs. KL-6, Krebs von den Lungen-6; HCs, healthy controls; EWs, exposed workers; SSs, simple silicosis subjects; CSs, complicated silicosis subjects; AUC, Area under the ROC curve; PPV, positive predictive value; NPV, negative predicted value.

ROC curve analysis aimed to identify cut-off values for (1) the diagnosis of fibrosis in silica-exposed subjects and (2) progression of pulmonary fibrosis in silicosis patients. As illustrated in Figure 2, the area under the curve (AUC) for serum KL-6 was notably larger in the CSs group compared to EWs (0.971, *p* < 0.001) and SSs (0.831, *p* < 0.001) groups. The best positive and negative predictive values (95.45 and 90.90% respectively) were obtained for differentiating exposed workers and silicosis patients with PMF.

### Analysis of predictors of silicosis in silica-exposed workers

Univariate and multivariate analyses were carried out to test whether serum KL-6, in addition to other covariates [age, gender, smoking history, body mass index (BMI), FVC % predicted, serum CRP and LDH, and Neutrophil-to-lymphocyte ratio (NLR)], is a predictor of silicosis onset in silica-exposed workers, as shown in Table 3a. The number of cases available in the analysis was 72, of which 57 events and 15 censored cases. Univariate analysis showed no significant predictors except age (hazard ratio (HR): 0.936; *p* = 0.001) and body mass index (BMI) (HR: 1.059; *p* = 0.036). The multivariate analysis showed that female gender was tendentially associated with a 30% increase in the risk of disease occurrence (*p* = 0.055).

**Table 3:** Univariate and multivariate COX proportional analysis for (a) silicosis occurrence and (b) silicosis progression.

	No. of events/total	Hazard ratio	95% CI	*p*-value
(a) Silicosis occurrence				
Univariate analysis				
Age years (continuous)	64/82	0.936	0.900–0.974	0.001
Gender (ref. male)	64/82	1.521	0.929–2.491	0.095
Smoking history (ref. smoker)	64/82	0.885	0.540–1.448	0.626
BMI kg/m^2^ (continuous)	62/80	1.059	1.003–1.118	0.037
FVC % pred (continuous)	61/78	1.001	0.988–1.014	0.896
CRP mg/L (continuous)	64/80	1.006	0.978–1.035	0.675
LDH U/L (continuous)	60/76	0.999	0.993–1.005	0.693
NLR ratio (continuous)	64/80	1.044	0.856–1.274	0.667
KL-6100 U/mL (continuous)	64/82	1.002	0.904–1.111	0.966
Multivariate analysis	57/72 (cumul.)			
Age years (continuous)		0.912	0.867–0.958	<0.001
Gender (ref. male)		1.699	0.989–2.919	0.055
BMI kg/m^2^ (continuous)		1.092	1.030–1.158	0.003
(b) Silicosis progression				
Univariate analysis				
Age years (continuous)	23/84	0.912	0.842–0.987	0.022
Gender (ref. male)	23/84	0.511	0.189–1.384	0.187
Smoking history (ref. smoker)	23/84	2.358	0.926–6.004	0.072
BMI kg/m^2^ (continuous)	23/82	0.906	0.821–1.000	0.050
FVC % pred (continuous)	23/81	0.986	0.964–1.010	0.248
CRP mg/L (continuous)	23/84	0.964	0.868–1.071	0.497
LDH U/L (continuous)	21/78	1.003	0.993–1.013	0.585
NLR ratio (continuous)	23/84	1.242	0.978–1.579	0.076
KL-6100 U/mL (continuous)	23/84	1.125	1.036–1.221	0.005
Multivariate analysis	21/75 (cumul.)			
Age years (continuous)		0.907	0.836–0.984	0.018
Gender (ref. male)		0.362	0.122–1.076	0.067
Smoking history (ref. smoker)		0.126	0.029–0.539	0.005
KL-6100 U/mL (continuous)		1.197	1.069–1.340	0.002

Data are presented as hazard ratios, representing the relative risk of developing (a) disease occurrence and (b) disease progression. Backward stepwise (Likelihood Ratio) analysis was performed. BMI, body mass index; FVC, forced vital capacity; CRP, C-reactive protein; LDH, Lactate dehydrogenase; NLR, Neutrophil-to-lymphocyte ratio; KL-6, Krebs von den Lungen-6; 95% CI, 95% Confidence interval.

### Analysis of predictors of PMF in silicosis subjects

**Figure 3:**
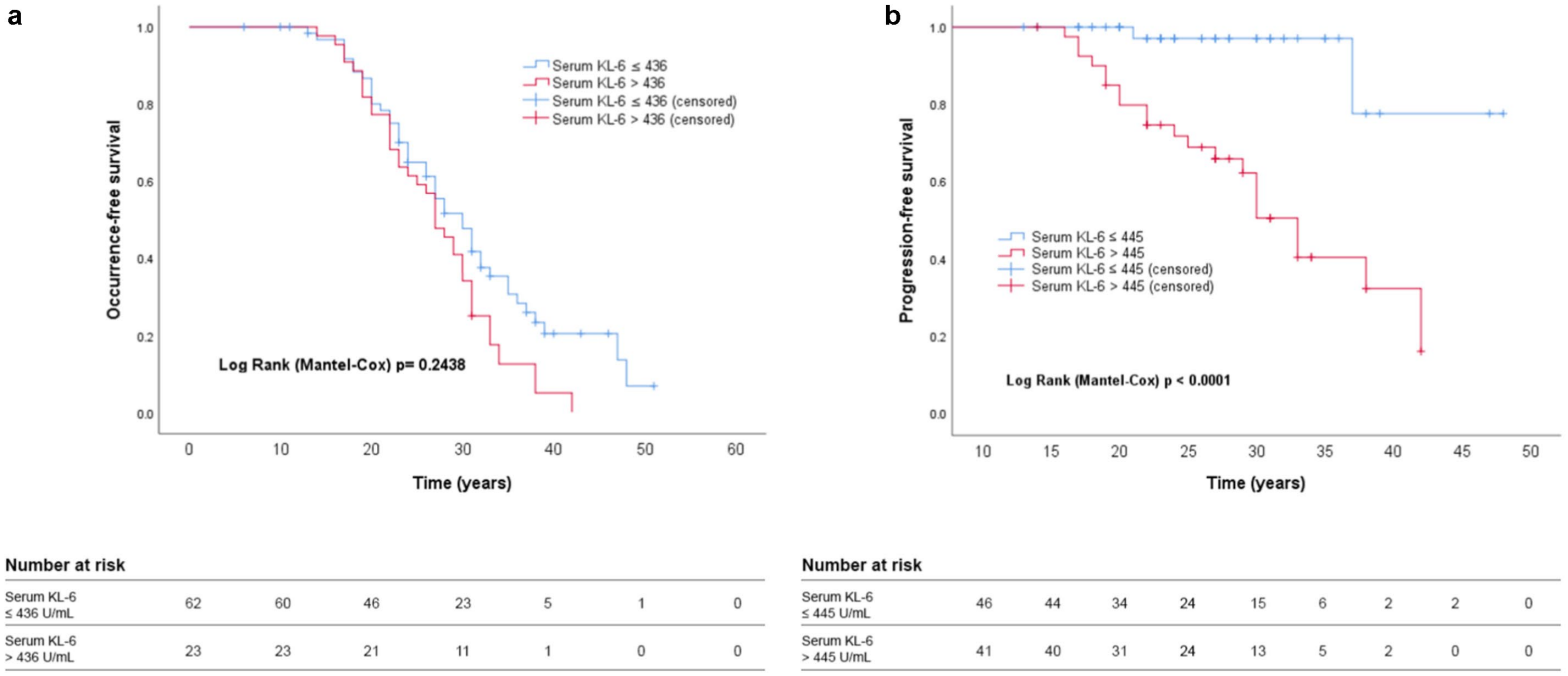
Kaplan–Meier analysis showing **(a)** silicosis occurrence and **(b)** silicosis progression according to serum KL-6 levels. The respective cut-off levels and significance (Log rank *p*) are shown in the graphic.

Kaplan–Meier analysis indicated a predictive value of serum KL-6 for disease progression in silicosis subjects (Figure 3b), but not for the silicosis occurrence (Figure 3a). At a latency time of 30 years, the subjects with KL-6 > 445 U/mL had a worse progression-free survival rate (51%) compared to those with KL-6 levels ≤ 445 U/mL (97%).

Table 3b shows the results of the univariate and multivariate analysis to identify PMF predictors. The number of cases available in the analysis was 75, of which 21 events and 54 censored cases. In the multivariate analysis, serum KL-6 levels were independently associated with PMF after adjustment for age, gender, smoking history, BMI, FVC % predicted, serum CRP and LDH, and NLR as covariates (HR 1.197; *p* = 0.002). Female gender and a negative smoking history were protective against PMF development.

## Discussion

Our study found higher serum KL-6 levels in patients with complicated silicosis with progressive massive fibrosis (CSs) compared to exposed workers without radiological signs of silicosis (EWs) and those with simple silicosis (SSs). We identified a cut-off value of serum KL-6 for predicting the progression of pulmonary fibrosis in silicosis patients. Differentiation between simple (opacities ≤ 1 cm) and complicated silicosis (opacities > 1 cm) was achieved at a serum KL-6 cut-off of 445 U/mL, with a specificity and sensitivity exceeding 90%. As with ILDs patients, our data indicated that KL-6 serum levels correlate with the severity of fibrosis assessed by standard radiology (25), supporting the use of KL-6 as biomarker for the lung fibrosis progression. Thus, the increased KL-6 serum levels in complicated and progressive forms could be explained by the alteration of the alveolar-capillary membrane due to extensive pulmonary fibrosis. These findings align with other studies linking KL-6 serum levels to disease progression in ILDs and IPF (13, 26).

Different cut-off values for serum KL-6 levels were proposed in the literature (27). One study suggests a cut-off of 500 U/mL to differentiate ILDs patients from healthy individuals (28). More than 70% of patients with different ILDs (idiopathic interstitial fibrosis, collagenosis, hypersensitivity pneumonitis, radiation pneumonitis, drug-induced pneumonitis, acute respiratory distress syndrome, sarcoidosis, alveolar proteinosis) had KL-6 serum levels exceeding 500 U/mL (26) and therefore, KL-6 cannot be considered a specific marker of silicosis. For patients with known exposure and already diagnosed small opacities in the lungs, the progression toward massive fibrosis has significant clinical implications and serum KL-6 might be of clinical relevance. In our study, a serum KL-6 cut-off of 436 U/mL differentiated EWs from those with PMF with a specificity and sensitivity exceeding 90%. However, KL-6 had an insufficient sensitivity (34.4%) to differentiate between EWs and SSs. Thus, based on these findings, we cannot propose serum KL-6 as a screening marker. However, the high specificity (95.2%) is a strong argument to consider serum KL-6 a marker of fibrosis, potentially serving as a valuable tool for monitoring the progression of pulmonary fibrosis in patients already diagnosed with silicosis. This approach is supported by experimental and clinical research, which demonstrate that exposure to crystalline silica leads to proliferation and hyperplasia of type II pneumocytes (29). Moreover, recent studies suggest that damaged alveolar epithelial cells release KL-6, which may promote the transformation of fibroblasts into myofibroblasts, increase collagen I and III production, and suppress HGF production (30). This highlights KL-6 as a contributor to fibrosis in various ILDs.

In CSs group, the level of KL-6 in serum was positively correlated with several inflammatory biomarkers, namely serum CRP and ESR. While extensive literature supports KL-6 as a marker of alveolar epithelial destruction and increased alveolar-capillary permeability (31), studies to correlate the serum KL-6 to inflammation are much less frequent. The CRP and the ESR were collected in the absence of an acute infection or other clinically manifest inflammatory conditions. In other conditions involving ILDs, such as those related to radiation treatment (32) or associated with connective tissue diseases (33), CRP or ESR levels were correlated with serum KL-6 in predicting disease severity. To the best of our knowledge, this study is the first to demonstrate a correlation between serum KL-6 levels and nonspecific inflammatory markers in patients with occupational progressive massive fibrosis, as expected considering the inflammatory background of fibrosis (34).

Another finding of this study was the negative correlation between KL-6 serum levels and lung function test results (VC % predicted and FVC % predicted), consistent with previous studies on ILDs of different causes (13, 26, 32, 35).

The multivariate analysis showed that serum KL-6 as continuous variable is independently associated with the disease progression in silicosis together with age, gender and smoking status. Higher levels of KL-6 were associated with shorter latency to PMF; subjects with serum KL-6 levels > 445 U/mL had a worse progression-free survival rate (51%) than those with serum KL-6 ≤ 445 U/mL (97%). These findings align with other studies linking serum KL-6 levels to the progression of pulmonary fibrosis in ILDs (36). Male gender has been also identified as an independent risk factor for progression in ILD in several studies (37) and smoking has been associated with a worse evolution in IPF (38). There is evidence that smoking directly is associated with fibrosis in the subpleural and peribronchiolar interstitium (39). In artificial stone dust silicosis, smoking seems to have a protective effect (40), but the mechanism of accelerated silicosis is different compared to the much less aggressive evolution of the chronic silicosis in the foundry workers. In a large cohort of foundry workers, smoking increased the risk of silicosis by 3.79 folds (41). To the best of our knowledge, there are no studies on foundry workers about the impact of smoking on the progression of silicosis. In this respect, the analysis of our patients encourages to prospectively investigate this potential influencer of the silicosis prognosis.

Although the results are promising, this study has potential limitations. First, it was a single-center study with a relatively small number of subjects in each subgroup. We are aware that this number is in the lower range of the estimated necessary numbers, but still remain significant for the long term follow up after the initial exposure (an average retention time exceeding 30 years across all subgroups). The group is rather homogenous in terms of occupational exposure to crystalline silica from cast iron foundries, a type of exposure which is rarely approached in European studies (42). In addition, the average age of our study population corresponds to exposure during their working time, a period in which the foundries had the same technology and protective measures. We therefore consider that the intensity of exposure was homogeneous and unlikely to have significantly influenced the results. Second, although the questionnaire used in this study complies with national regulations and is commonly used in occupational disease department practice, designed to collect basic demographic information, symptoms and data on occupational exposure to pneumoconiogenic dusts and fibers, its validity and reliability were not independently assessed for this study. Moreover, the diagnosis was based on the X-ray and not on CT or HR-CT, to more accurately assess the extension of the pulmonary fibrosis lesions. However, the current classification in clinical practice is based on X-ray, which remains the most extended screening method. Revisions of the X-rays by experienced physicians mitigates this risk, particularly the misclassification between SS and CS. We are aware that several other factors might be related to the level of serum KL-6, such as polymorphisms of MUC1 (43, 44) or the inflammatory/immunological status reflected in other serum biomarkers. To the best of our knowledge there is no information about the interaction between MUC genes expression and the KL-6 serum levels in silicosis; their potential influence might represent a limitation. We have checked on the relation between the complicated silicosis and the CRP, ESR fibrinogen, and LDH levels and found significant association with the progression of silicosis, even though in CS group both CRP and ESR were correlated with KL-6 serum levels. We did not check for other biomarkers such as serum amyloid A or surfactant protein –D, as previous publications found of no relation with the progression of silicosis (45, 46), not even in the stone benchtop industry workers, which have a more aggressive form of silicosis than the silicosis from foundries included in our analysis (47). Translating the lessons learnt from other interstitial lung fibrosis, a panel of biomarkers might also perform better for silicosis. Based on our results we can only support that the selection of biomarkers to characterize the PMF should include serum KL-6. Lastly, we did not monitored KL-6 serum concentrations over time and we cannot draw any conclusion on the prognostic value of KL-6.

## Conclusion

In conclusion, there is a scarcity of studies specifically investigating the correlation between serum KL-6 and exposure to pneumoconiogenic mineral dusts in European foundry workers. KL-6 is already approved as a diagnostic and prognostic biomarker for diffuse interstitial lung diseases in some countries, and its pathophysiologic mechanism supports a potential utility in pneumoconioses. From our results, serum KL-6 appears to be a promising biomarker of the progression of occupational fibrosis lesions in silica-exposed patients.

## Data Availability

The raw data supporting the conclusions of this article will be made available by the authors, without undue reservation.
