# Correction: Serum concentrations of Krebs von den Lungen-6 as prognostic biomarker in patients with silicosis

**DOI:** 10.3389/fmed.2025.1654729

**Published:** 2025-07-28

**Authors:** Raluca-Andreea Smǎrǎndescu, Marina-Ruxandra Oțelea, Eugenia Panaitescu, Andrä Jitka, Francesco Bonella, Agripina Rașcu

**Affiliations:** ^1^Carol Davila University of Medicine and Pharmacy, Clinical Department 5, Internal Medicine, Bucharest, Romania; ^2^Department of Occupational Medicine, Colentina Clinical Hospital, Bucharest, Romania; ^3^Carol Davila University of Medicine and Pharmacy, Department 3 - Complementary Sciences, Bucharest, Romania; ^4^Center for Interstitial and Rare Lung Disease, Department of Pneumology, Ruhrlandklinik University Hospital, University of Duisburg-Essen, Essen, Germany

**Keywords:** KL-6, silicosis, occupational fibrosis, crystalline silica, biomarker, disease progression

An incorrect **Funding** statement was provided. The correct funding statement reads:

The author(s) declare that financial support was received for the research and/or publication of this article. This work was supported by the University of Medicine and Pharmacy Carol Davila, through the institutional program Publish not Perish.

The **Acknowledgments** was not included. The Acknowledgments statement appears below:

Publication of this paper was supported by the University of Medicine and Pharmacy Carol Davila, through the institutional program Publish not Perish.

The original version of this article has been updated.

