# Supplementary material for: Serum concentrations of Krebs von den Lungen-6 as prognostic biomarker in patients with silicosis
Source: Front Med (Lausanne). 2025 Jun 6;12:1579209. doi: 10.3389/fmed.2025.1579209 (PMC12178848; doi:10.3389/fmed.2025.1579209)
Supplement: Supplementary file 1 [file Table_1.DOCX]

**Supplemental material**

**APPENDIX A**

**Abridged ILO classification for Silicosis (according to the International Labour Organization, Guidelines for the use of the ILO International Classification of Radiographs of Pneumoconioses, revised edition 20112022, Geneva)**

| Simple silicosis with  opacities (p, q, r) ≤ 10 mm | Complicated silicosis (progressive massive fibrosis) with opacities (A, B, C) > 10mm |
| --- | --- |
| p < 1,5mm | A - one opacity with longest dimension <50mm, or several large opacities with their sum of their longest dimension <50mm |
| q 1,5-3mm | B - one large opacity with the longest dimension > 50mm but < the equivalent of the right upper zone, or several large opacities with their sum of their longest dimension >50mm but < the equivalent of the right upper zone |
| r 3-10mm | C - one large opacity > the equivalent of the right upper zone, or several large opacities when combined > the equivalent of the right upper zone |

**APPENDIX B**

**Occupational and medical history questionnaire**

| Name: |  |
| --- | --- |
| Date of birth: |  |
| Gender: | Male/Female |
| Do you live next to or near an industrial plant? | Yes/No |
| Do you have a hobby or craft?  If yes, name them: | Yes/No |
| Smoking status: | Current smoker/Former smoker/ Non-smoker |
| Are you exposed to secondhand tobacco smoke? | Yes/No |
| Alcohol consumption: | Yes/No |
| Do you follow any diet? | Yes/No |
| Weight | kg |
| Height | m |
| Do you have any medical conditions and diseases?  If yes, name them: | Yes/No |
| Do you have any symptoms?  If yes, describe them: | Yes/No |
| Do you follow any treatment for a chronic condition? | Yes/No |
| Have you ever been advised to change jobs or work assignments because of any health problems or injuries? | Yes/No |
| Have you been exposed to dust or fibers? | Yes/No |
| Have you been exposed to crystalline silica? | Yes/No |
| Have you been diagnosed with silicosis?  If yes, mention the year you were diagnosed: | Yes/No |
| Do you know the names of the dusts or fibers you were exposed to?  If yes, name them: | Yes/No |
| Are your work clothes laundered at home? | Yes/No |
| Do you shower at work? | Yes/No |
| Do you use protective equipment (gloves or  respirator protectors)? | Yes/No |
| If you wear respirator protectors, how often do you change it (the mask) during a shift? | Once/Twice/ ≥ Thrice |
| Do you know of any co-workers experiencing similar or unusual symptoms? | Yes/No |
| Do your symptoms seem to be aggravated by a specific activity? | Yes/No |
| Occupational profile:  Job title  Job industry  Date job began  Are you still working in this job?  If no, date job ended? |  |
| Describe the technological process |  |
| Has the technological process improved over time? | Yes/No |
| Is there efficient ventilation in your workplace? | Yes/No |

**APPENDIX C**

**Correlations between KL-6 (Krebs von den Lungen-6), CRP (C-reactive protein), ERS (erythrocyte sedimentation rate), VC % predicted (vital capacity), and FVC % predicted (forced vital capacity) in CSs group**

**
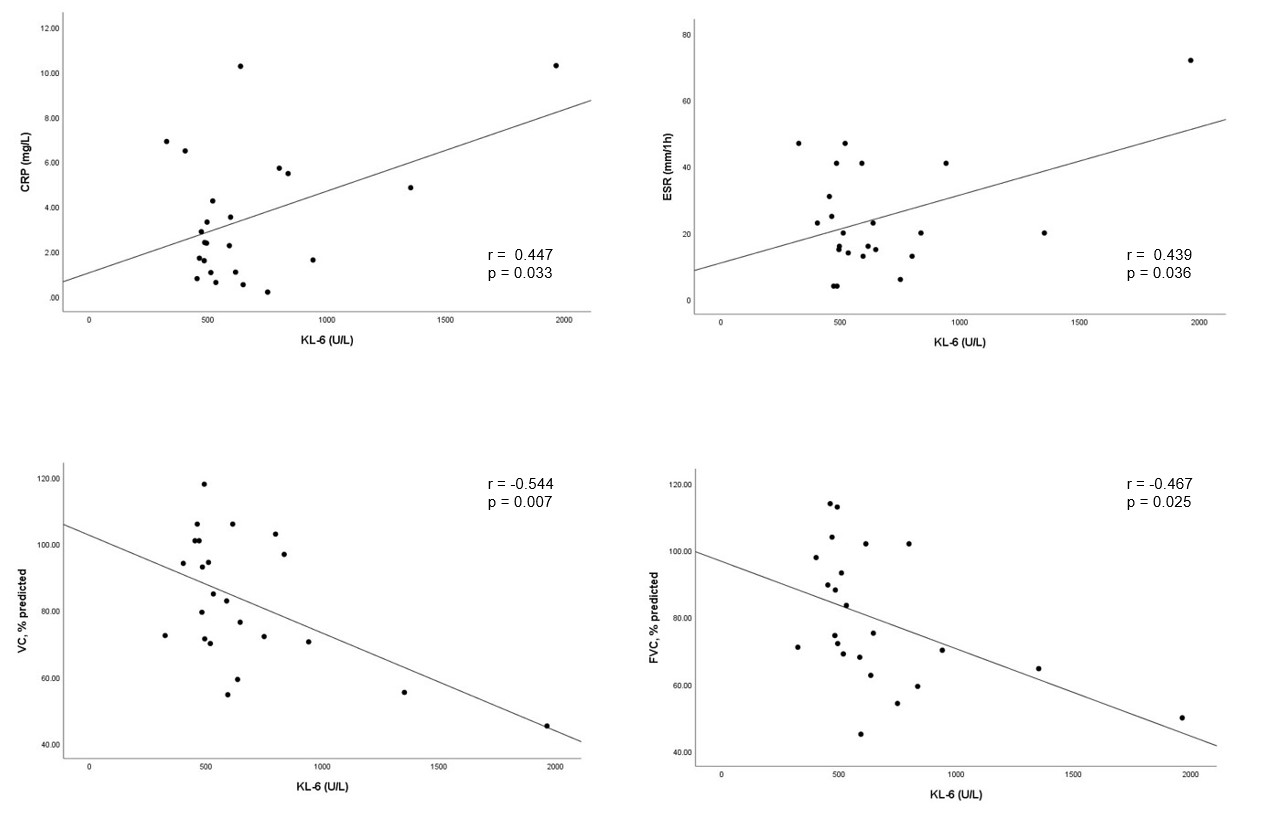
**
